# A functional applied material on recognition of metal ion zinc based on the double azine compound

**DOI:** 10.1016/j.tet.2017.04.001

**Published:** 2017-05-18

**Authors:** Taibao Wei, Guoyan Liang, Xiaopeng Chen, Jin Qi, Qi Lin, Youming Zhang, Hong Yao

**Affiliations:** a Key Laboratory of Eco-Environment-Related Polymer Materials, Ministry of Education, Gansu 730070, PR China; b Key Laboratory of Polymer Materials, College of Chemistry and Chemical Engineering, Northwest Normal University, Lanzhou, Gansu 730070, PR China

**Keywords:** Fluorescent probe, Double azine, Fluorescence switch, Test strip

## Abstract

A colorimetric and fluorescent probe **L** has been designed and synthesized, which bearing the double azine moiety and showing a detection limit of 2.725 × 10^−7^ M towards Zn^2+^. Based on the basic recognition mechanism of ESIPT and CHEF effect, the **L** has high selectivity and sensitivity to only Zn^2+^ (not Fe^3+^, Hg^2+^, Ag^+^, Ca^2+^, Co^2+^, Ni^2+^, Cd^2+^, Pb^2+^, Cr^3+^, and Mg^2+^) within the physiological pH range (pH = 7.0–8.4) and showed a fluorescence switch. Moreover, this detection progress occured in the DMSO/H_2_O ∼ HEPES buffer (80/20, v/v; pH 7.23) solution which can conveniently used on test strip.

## Graphical abstract

A colorimetric and fluorescent probe **L** has been designed and synthesized, bearing the double azine moiety and showing a detection limit of 2.725 × 10^−7^ M towards Zn^2+^. Based on the basic recognition mechanism of ESIPT and CHEF effect, the probe owes high selectivity and sensitivity to only Zn^2+^ (not Fe^3+^, Hg^2+^, Ag^+^, Ca^2+^, Co^2+^, Ni^2+^, Cd^2+^, Pb^2+^, Cr^3+^, and Mg^2+^) within the physiological pH range (pH = 7.0–8.4) and showed a fluorescence switch. Moreover, this detection progress occur in the DMSO/H_2_O ∼ HEPES buffer (80/20, v/v; pH 7.23) solution which can conveniently used on test strip, and it can anti interference of others.

**Figure:**
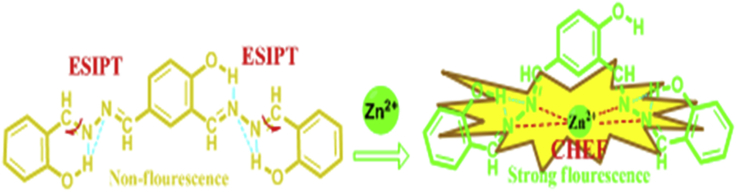

## 1 Introduction

Due to the effects of metal ions in the environment and human health, there are many selective chemo probes for these metal ions have been developed.^1^ Living system and natural human health need quantitative intakes of most of the metal ions including heavy metal ions.^2^, ^3^, ^4^ Zinc is one of the essential trace metal elements in the human body like ferrum^5^ and copper,^6^ which occupies an important position in various chemical, environmental and physiological systems. Although the majority of the biological zinc ions are tightly sequestered by proteins, free Zn^2+^ pools containing large amounts of Zn^2+^ exist in certain tissues, for example, high concentrations up to 0.1–0.5 mM of Zn^2+^ have been reported in brain tissues.^7^ Moreover, the unregulated zinc level in the body may lead to a number of severe neurological diseases (e.g. Alzheimer's disease and epilepsy).^8^, ^9^, ^10^, ^11^ Thus, the detection and recognition for the metal ion zinc are necessary, and we need various convenient methods or chemical molecules to realize it. For example, Yang Wei and his coworkers^12^ synthesized a Europium-based luminescent chemo probes for Zn^2+^ with quinoxaline; and Ajay Misra's group constructed a hydrazine to detect Zn^2+^^13^. And it is imperative to search for more practical fluorescent probes for selective detection of zinc.

Field application requires the techniques which process wonderful selectivity, speedy sensitivity, consistency, and exclusively easy operation. Various methods have been reported to detect both the metal ions and anions such as atomic absorption spectroscopy,^14^ inductively coupled plasma atomic emission spectrometry^15^ and electrochemical methods.^16^ This techniques employ convenient approaches to realize more accurate detection. And several methods require tedious sample preparation procedures, sophisticated instrumentation and trained operators. Fluorescence technology provides a convenient and ordinary method in the context of sensing of environmentally and biologically pertinent metal ions.^17^ The literature reported one kind of fluorescent probes is composed of azine compounds.

Azine is a special kind of conjugate schiff base, which is a general term for unsaturated six membered heterocyclic or heteroatom compounds containing one or more nitrogen atoms. There are many kinds of compounds which process some structural similarity to the Azine compounds, such as pyridine, pyrimidine, triazine and thiophene, have being attached attentions in recent years.^18^

The structures of nitrogen atoms which contain lone pair electrons, can lead to the formation of coordination effect with many metals owing their outer space orbits. Supplemented by the ortho-oxygen and sulfur atoms on this kind of compounds, the research on it complied in the field of fluorescence detection becomes more and more active.^19^ Azine, as a kind of fluorescence probe, contains great opportunity of sensitivity, selectivity, real-time and in situ detection effect.^20^ On the other hand, Azine compounds as a kind of organic molecules, process great advantages in the fields of molecular design and structural characterization. And can employ its own distinctive chemical bonds, coordination bonds and unique spatial molecular conformation to selectively bind with specific metal ions, which shows high recognition ability and have attracted great interest of researchers.^21^ According to these researches have been made, we synthesized a dialdehyde azine compound (**L**), which showed great opportunity of sensitivity and selectivity in the progress of detecting the metal ion Zn^2+^.

## 2 Results and discussion

The synthesis course of chemical probe **L** was depicted in Scheme S1 and operation details were described in experimental section in the Supporting information. It was fully characterized by spectroscopic analysis and mass-spectrography. The ^1^H NMR and ^13^C NMR (Figs. S1–S6) spectra were used to confirm the structure and the purity of the probe. The ESI−MS (Fig. S7) spectrum showed the major peak at *m*/*z* 385.1021 [M−H]^−^, which perfectly matched the estimated molecular weight of [C_22_H_17_N_4_O_3_- H]^−^. IR (Fig. S8) spectrum for the molecule **L** showed a vibration band at 1654 cm^−1^ assigned to stretching vibrational mode of imine (-CH=N-) groups and a broad peak at 3469 cm^−1^ assigned to stretching vibrational mode of hydroxyl (-OH) groups.

**Fig. 1:**
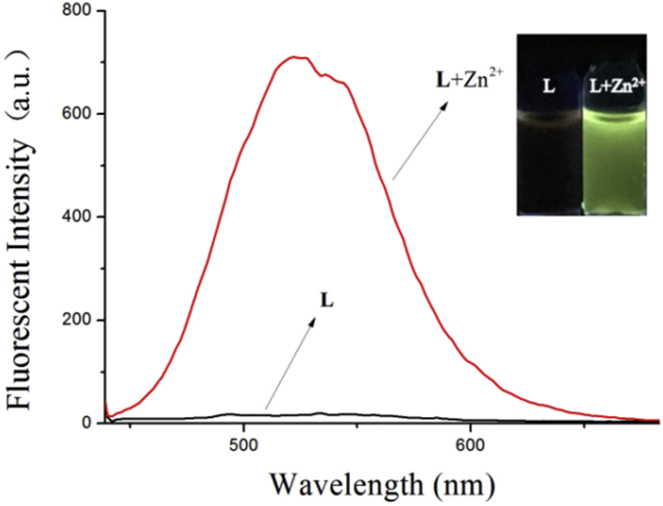
Fluorescence spectra of the probe **L** (2 × 10^−5^ mol L^−1^) with adding Zn^2+^ in DMSO/H_2_O ∼ HEPES buffer (8/2, v/v; pH = 7.23) solution. Inset: color changes of **L** and with Zn^2+^.

In the fluorescence experiments of the probe **L** responsing to Zn^2+^, we selected the DMSO/H_2_O ∼ HEPES buffer (80/20, v/v; pH 7.23) as solution for the target of excluding the possibility in fluence of pH fluctuation. As shown in Fig. 1, the **L** had no fluorescent nature with the excitation wavelength was at 425 nm, but an unique and new emission peak appeared at 520 nm when added 20 equiv. of Zn^2+^ into the solution of **L,** and the color of this complex shew fluorescently green.

**Fig. 2:**
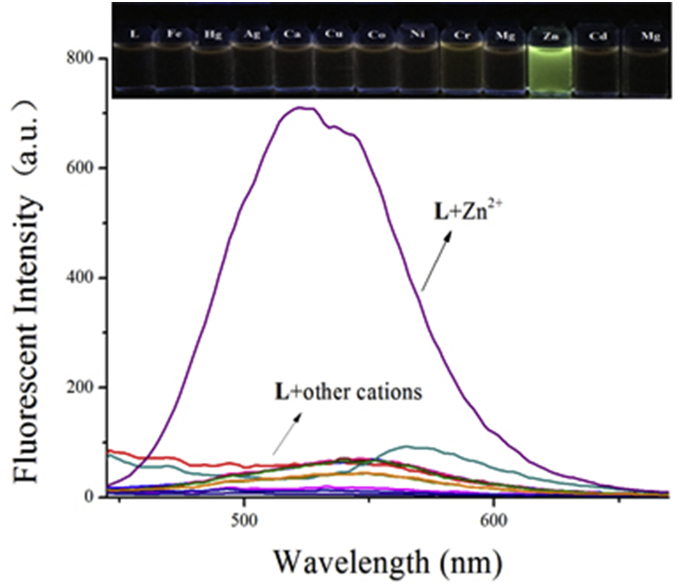
Fluorescence spectra of probe **L** (2 × 10^−5^ mol L^−1^) with various cations (Fe^3+^, Hg^2+^, Ag^+^, Ca^2+^, Cu^2+^, Co^2+^, Ni^2+^, Cd^2+^, Pb^2+^, Zn^2+^, Cr^3+^ and Mg^2+^) in DMSO/H_2_O ∼ HEPES buffer (80/20, v/v; pH = 7.23) solutions. Inset: color changes of **L** with various anions.

To find the response property of the probe **L** to other various cations, such as Fe^3+^, Hg^2+^, Ag^+^, Ca^2+^, Cu^2+^, Co^2+^, Ni^2+^, Cd^2+^, Pb^2+^, Cr^3+^ and Mg^2+^, we added these cations according to the priority to DMSO/H_2_O ∼ HEPES buffer (80/20, v/v; pH 7.23) solutions of probe **L** no significant color or spectrum changes were observed (Fig. 2). Which Suggested that the probe **L** was a special recognition subject for Zn^2+^.

**Fig. 3:**
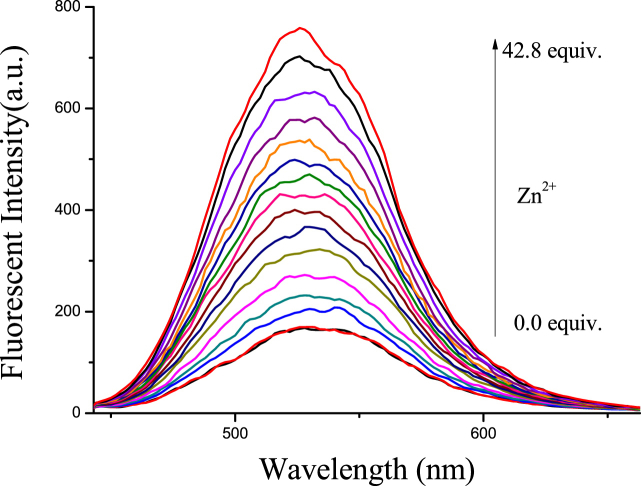
Fluorescence spectral changes of **L** (c = 2 × 10^−5^ M) in the presence of different concentrations of Zn^2+^ ions in DMSO/H_2_O ∼ HEPES buffer (80/20, v/v; pH = 7.23) solutions.

**Fig. 4:**
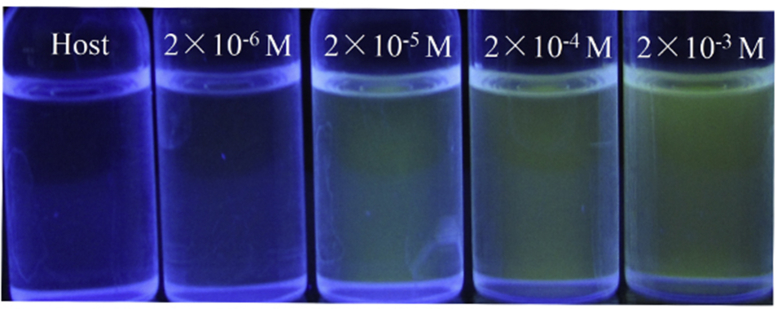
Naked-eye detection limit under an UV lamp at 365 nm. From left to right, the concentration of Zn^2+^ were 0; 2.0 × 10^−6^ mol L^−1^; 2.0 × 10^−5^ mol L^−1^, 2.0 × 10^−4^ mol L^−1^, and 2.0 × 10^−3^ mol L^−1^.

The probe **L** could act as a functional material for the detection of Zn^2+^ in DMSO/H_2_O ∼ HEPES buffer (80/20, v/v; pH = 7.23) solutions. For instance, only Zn^2+^ (4.0 × 10^−4^ mol L^−1^) could open the fluorescence emission of **L,** not other various ions when adding them into 2 × 10^−5^ mol L^−1^ solutions of **L**. Moreover, as shown in Fig. 3, the fluorescence emission at 520 nm increased when gradually added the Zn^2+^ into this solution of probe. The detection limit of **L** to Zn^2+^ is estimated to be 2.725 × 10^−7^ M. Meanwhile, the fluorimetric detection limit of Zn^2+^ by the naked eye for probe **L** was also tested. As shown in Fig. 4, under an UV lamp at 360 nm the minimum concentration of Zn^2+^ for the fluorescence color change, observed by the naked eye was 2.0 × 10^−5^ M.

**Fig. 5:**
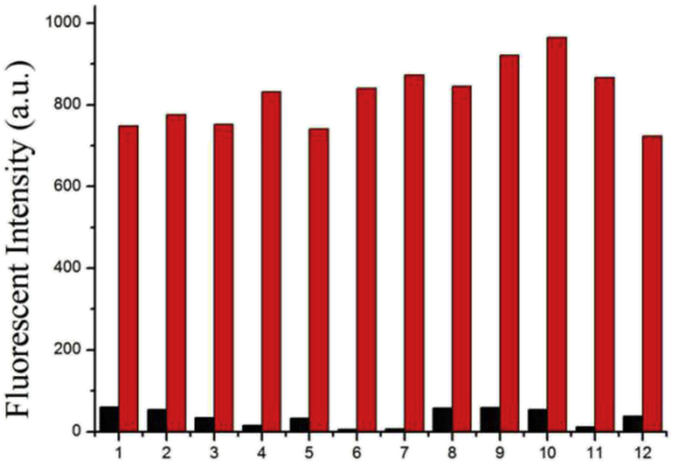
Fluorescence intensity changes of the **L** (20 μM) to Zn^2+^ (20 equiv.) in the presence of various test cations (20 equiv.) in DMSO/H_2_O ∼ HEPES buffer (80/20, v/v; pH = 7.23) solutions. Key: left to right, (1) only **L**, **L** + Zn^2+^, (2) **L** + Fe^3+^, **L** + Zn^2+^+ Fe^3+^; (3) **L** + Hg^2+^, **L** + Zn^2+^ + Hg^2+^; (4) **L** + Ag^+^, **L** + Zn^2+^ + Ag^+^; (5) **L** + Ca^2+^,**L** + Zn^2+^ + Ca^2+^; (6) **L** + Cu^2+^, **L** + Zn^2+^ + Cu^2+^; (7) **L** + Co^2+^, **L** + Zn^2+^ + Co^2+^; (8) **L** + Ni^2+^, **L** + Zn^2+^ + Ni^2+^; (9) **L** + Cd^2+^, **L** + Zn^2+^ + Cd^2+^; (10) **L** + Pb^2+^, **L** + Zn^2+^ + Pb^2+^; (11) **L** + Cr^3+^, **L** + Zn^2+^ + Cr^3+^ and (12) **L** + Mg^2+^, **L** + Zn^2+^ + Mg^2+^.

Although the probe **L** exhibited the single colorimetric and fluorescent recognition ability for Zn^2+^, the ability of detecting metal cations selectively over other competing metal cations was an essential aspect for many prospective chemical probes. In order to utilize compound **L** as a Zn^2+^ ion-selective fluorescence probe, competition experiments were made at the presence of Zn^2+^ (10 μM) mixed with 10 μM of another cations. As shown in Fig. 5, there was no significant interference when we used the probe **L** to detect Zn^2+^ at the presence of many competitive metal cations and all of the solutions containing Zn^2+^ shew green under an UV lamp at 360 nm. The results showed that the complex state of the probe **L** with Zn^2+^ was almost unaffected by the exists of other cations.

**Fig. 6:**
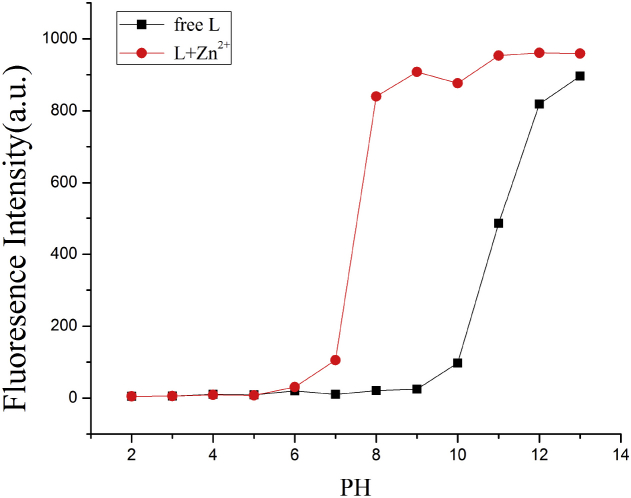
PH value affected the charge distribution of receptor **L** and **L** + Zn^2+^.

Since the charge distribution of the probe **L** could be influenced by pH value and its inherent fluorescence properties could be changed within a range of pH fluctuation. The response property of probe **L** were discussed by adding a certain volume HEPES buffered solutions with various pH values into the DMSO solution of **L**, respectively (pH ranged from 2.0 to 13.0, the step-size was 1.0 pH unit). As clearly showed in Fig. 6, the complex of **L**-Zn^2+^ demonstrated a significant fluorescence response between pH 7 and 11, including the physiologically relevant range of pH 7.0–8.4. These results suggested that Zn^2+^ could be clearly detected by the fluorescence spectral measurement using **L** within the physiological pH range (pH = 7.0–8.4)^22^ and this properties made **L** act as a pH controlled alkalescent fluorescent switch.

**Fig. 7:**
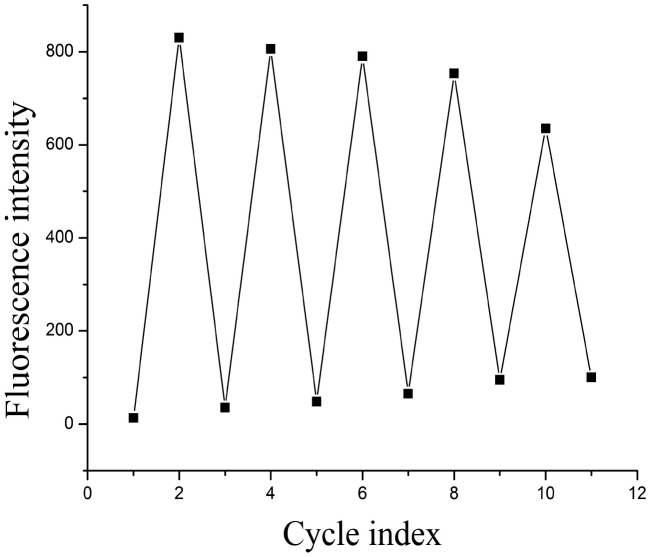
Fluorescence intensity absorption switching cycles of **L** (2 × 10^−5^ mol L^−1^) controlled by alternating addition of EDTA and Zn^2+^ in DMSO/H_2_O (v/v, 80/20).

Better applicability needed kinds of methods to be confirmed. The addition of EDTA to the probe **L** showed that the process of titrating probe **L** with EDTA was reversible, and the reversible process could be repeated at least five times (Fig. 7).

**Fig. 8:**
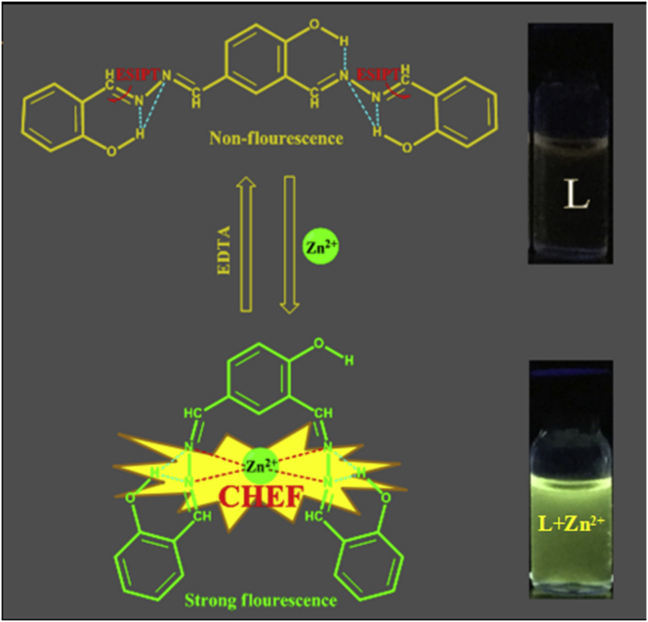
A possible sensing mechanism of the probe **L** to Zn^2+^.

On account of this, it was notable that the probe **L** not only could be treated as a ‘‘turn-on’’ probe for Zn^2+^ but successfully differentiated Zn^2+^ from Cd^2+^, which had always been a difficult problem to solve in the past.^23^, ^24^ In order to accurately study the specific reasons for the opening of fluorescence of the probe **L**, the ^1^H NMR titration experiments were carried out in the d_6_-DMSO at the concentration of 15 mM when encountered the ion Zn^2+^. As specially marked and showed in Fig. S9, the chemical peaks for protons located at the phenolic hydroxyl gradually shift upfield on NMR time scale when the Zn^2+^ were added quantificationally, and the chemical peaks of protons on the hydrazone slightly shifted downfield simultaneously. These result suggested that the cloud density around the phenolic hydroxyl proton changed from relatively small to large because of the weakening of N—H hydrogen bonds, and this weakening was caused by the coordination effect between the outer lone pair electrons on nitrogen atoms and the space orbits on zinc cations. And it was correlative with the preferential fluorescence enhancement for Zn^2+^ which might be caused by the formation of a chelate complex (rigid system) between **L** and the Zn^2+^ because of the chelation-enhanced fluorescence (CHEF) effect.^25^ Additionally, compound **L** was originally poor fluorescent due to the two possible effects: (Ι) the isomerization of —HC=N– double bonds occur in the excited state^26^ and (Π) excited-state intramolecular proton transfer (ESIPT) (see Fig. 8).^27^, ^28^, ^29^, ^30^, ^31^, ^32^ And the ESI-MS spectrum for the strong florescent complex also shown an obvious peak at m/z 452.3257 assignable to [**L** + Zn^2+^ + H^+^](Fig. S10).

Based on the favorable features of the probe **L** in solution, the test strips were prepared by immersing filter papers into the solutions of probe **L** (c = 1 × 10 ^−3^M) and then drying them in air to determine the practicability of a ‘dip-stick’ method for the detection of Zn^2+^.

**Fig. 9:**
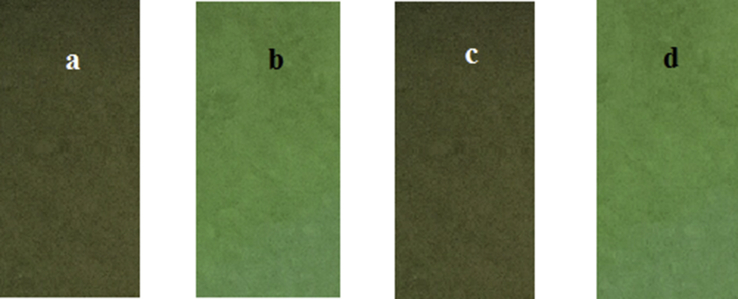
Fluorescence change of the test strips of **L** (a. Only **L** (1 × 10^−3^ M); (b. after immersion into DMSO/H_2_O ∼ HEPES buffer (80/20, v/v; pH = 7.23) solutions with Zn^2+^; (c. after immersion into DMSO/H_2_O ∼ HEPES buffer (80/20, v/v; pH = 7.23) solutions with Cd^2+^; (d. after immersion into DMSO/H_2_O ∼ HEPES buffer (80/20, v/v; pH = 7.23) solutions with Zn^2+^ and Cd^2+^ under irradiation at 254 nm. Among them: test strips selectively fluorimetric and colorimetric detect.

When the test strips coated with the probe **L** were immersed into the DMSO/H_2_O ∼ HEPES buffer (80/20, v/v; pH = 7.23) solutions of Zn^2+^, obvious fluorescent enhancement appeared (Fig. 9a and b). Similarly to detection of metal cations selectively over other competing metal cations, we prepared the test strips of the probe **L** with Cd^2+^ in the DMSO/H_2_O ∼ HEPES buffer (80/20, v/v; pH = 7.23) solution to compare with the test strips of **L** with Zn^2+^ and Cd^2+^. The test result demonstrated the preference of the **L** toward Zn^2+^ over the Cd^2+^ as shown in Fig. 9 (c and d). It was imperative to note that the chemical probe **L** for Zn^**2+**^ did not have any interference from the Cd^2+^. Moreover, fluorescence titration spectra of **L** (c = 2 × 10^−5^ M) in the presence of different concentrations of Cd^2+^ ions was made (Fig. S11). It can be clearly find that although the quantity of Cd^2+^ reached 20 equiv. to **L**, the intensities of solutions almost had no change, and the highest intensity was only 20 (a.u.). Generally, it was difficult to distinguish Zn^2+^ from Cd^2+^ in common solution owing to the Cd^2+^ and Zn^2+^ had analogous possessions and caused a strong interference.^33^ Therefore, the chemosensor **L** demonstrated a noteworthy propensity to discriminate Zn^2+^ from Cd^2+^ in a given solution. The development of such a ‘dip-stick’ method was extremely attractive for ‘in-the-field’ measurements that did not require any additional equipment. Therefore, the test strips of **L** had excellent application value in the detection of Zn^2+^.

Besides, in order to obtain the fluorescence quantum yields for both **L** and **L** with Zn^2+^, we used Rhodamine-B (c = 2 × 10^−6^ M) as a reference standard sample to figure out these values. Finally, the fluorescence quantum yields for **L** and **L** with Zn^2+^ were concluded to be 8.94% and 83.6% respectively.

In summary, herein we have synthesized and characterized a new simple and inexpensive fluorescent probe **L** which contained double azine moiety. It always exhibited swift and wonderful sensitivity toward Zn^2+^ through a palpable colorimetric and turn-on fluorescence response with the detection limit towards Zn^2+^ was 2.725 × 10^−7^ M. The novel chemical probe **L** not only had a very good practicality in the living body, but also had a significant fluorescent open repeatability with EDTA. The further study for fluorescent probe needs correlative support such as this kind of azine derivatives and we wish great development on it would be made.

## 3 Experimental section

### 3.1 Materials and methods

Fresh doubly distilled water was used throughout the experiment. All other reagents and solvents were commercially available at analytical grade and were used without further purification. ^1^H NMR and ^13^C NMR spectra were recorded on a Mercury-400BB spectrometer at 400 MHz for ^1^H. The chemical shifts, reported in ppm downfield from tetra spectra, were recorded with a Mercury-400BB spectrometer at 400 MHz (TMS, d scale with the solvent resonances as internal standards). Electrospray ionization mass spectra (ESI-MS) were measured on an Agilent 1100 LC-MSD-Trap-VL system. UV–visible spectra were recorded on a Shimadzu UV-2550 spectrometer. The photoluminescence spectra were performed on a Shimadzu RF-5301 fluorescence spectrophotometer. The melting points were measured on an X-4 digital melting-point apparatus (uncorrected). The infrared spectra were performed on a Digilab FTS-3000 FT-IR spectrophotometer.

### 3.2 General procedure for spectroscopy

Fluorescence spectroscopy was carried out in a DMSO/H_2_O ∼ HEPES buffer (80/20, v/v; pH 7.23) solution on a Shimadzu RF-5301 spectrometer. Any changes in the fluorescence spectra of the synthesized compound were recorded upon the addition of perchlorate salts while keeping the ligand concentration constant (2.0 × 10^−5^ M) in all experiments. The perchlorate salts of the ions (Fe^3+^, Hg^2+^, Ca^2+^, Cu^2+^, Co^2+^, Ni^2+^, Cd^2+^, Pb^2+^, Zn^2+^, Cr^3+^, and Mg^2+^) were used for the fluorescence experiments.

For ^1^H NMR titrations, the solution of **L** and Zn^2+^ were prepared in DMSO-*d*_6__._ Aliquots of the solutions were mixed directly in the NMR tubes d_6_-DMSO.
