# Supplementary material for: A functional applied material on recognition of metal ion zinc based on the double azine compound
Source: Tetrahedron. 2017 May 18;73(20):2938–42. doi: 10.1016/j.tet.2017.04.001 (PMC5407350; doi:10.1016/j.tet.2017.04.001)
Supplement: Supplementary file 1 [file mmc1.doc]

Supporting Information

*Key Laboratory of Eco-Environment-Related Polymer Materials, Ministry of Education of China; Key Laboratory of Polymer Materials of Gansu Province; College of Chemistry and Chemical Engineering, Northwest Normal University, Lanzhou, Gansu, 730070, P. R. China*

1. Synthetic procedure of chemical probe **L**.

***Scheme S1.*** Synthetic routes to **L**.

**b** A solution of salicylaldehyde (5.0 ml, 47.0 mmol), 37% aqueous solution of formaldehyde (3.6 ml, 50.0 mmol) and concentrated hydrochloric acid (50 mL) stirred in a dry and packaged round flask at the room temperature until a large number of white solids separated out. Then the ether solution of these solid needed to be washed by 10% NaOH solution to made the pH be 7~8 and then filtered out the water in this mixture. After that, the ether solution was evaporated under reduced pressure and we gained white solid powder which was then recrystallized by petroleum ether and the white needle crystal 5-chloromethyl salicylaldehyde **b** was gained. m.p = 84-86℃.^1^H NMR (600 MHz, *d*_6_-DMSO) δ 10.89 (s, 1H), 10.24 (s, 1H), 7.69 (d, J = 2.4 Hz, 1H), 7.57 – 7.52 (m, 1H), 6.99 (d, J = 8.5 Hz, 1H), 4.73 (s, 2H). ^13^C NMR (151 MHz, *d*_6_-DMSO), 191.22 , 161.17 , 137.34 , 129.60 , 122.59 , 118.18 , 46.15 .


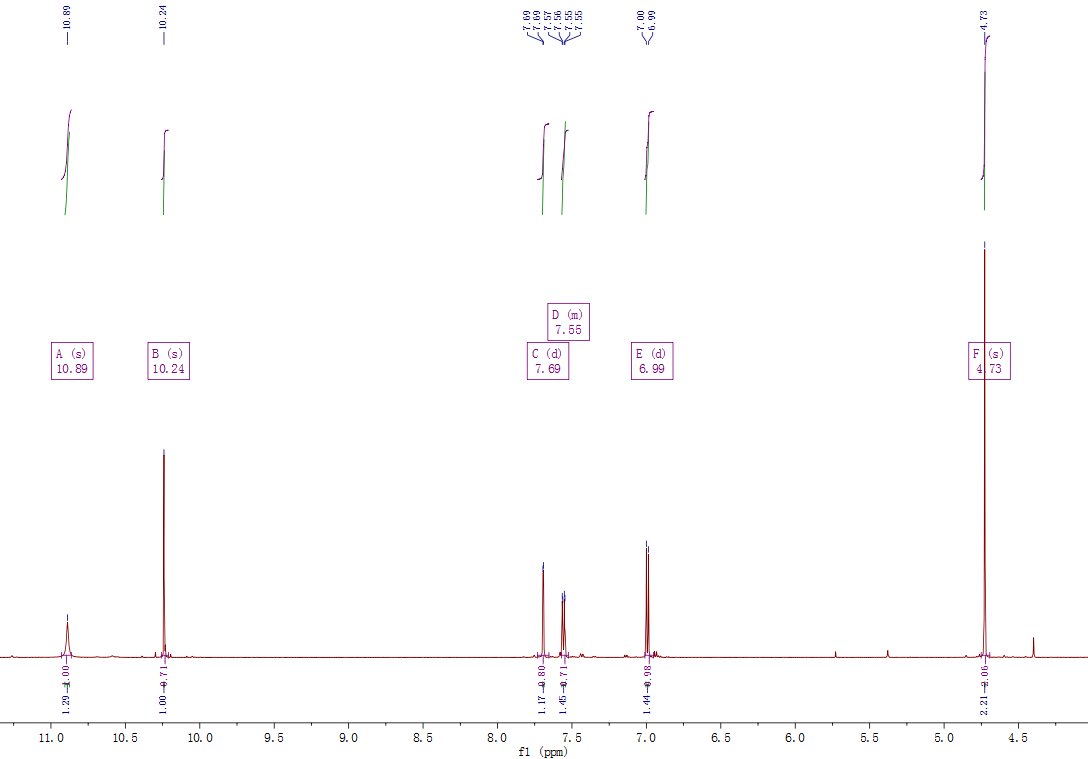


**Fig. S1** ^1^H NMR spectrum (600 MHz, d_6_-DMSO, 293 K) of **b**.


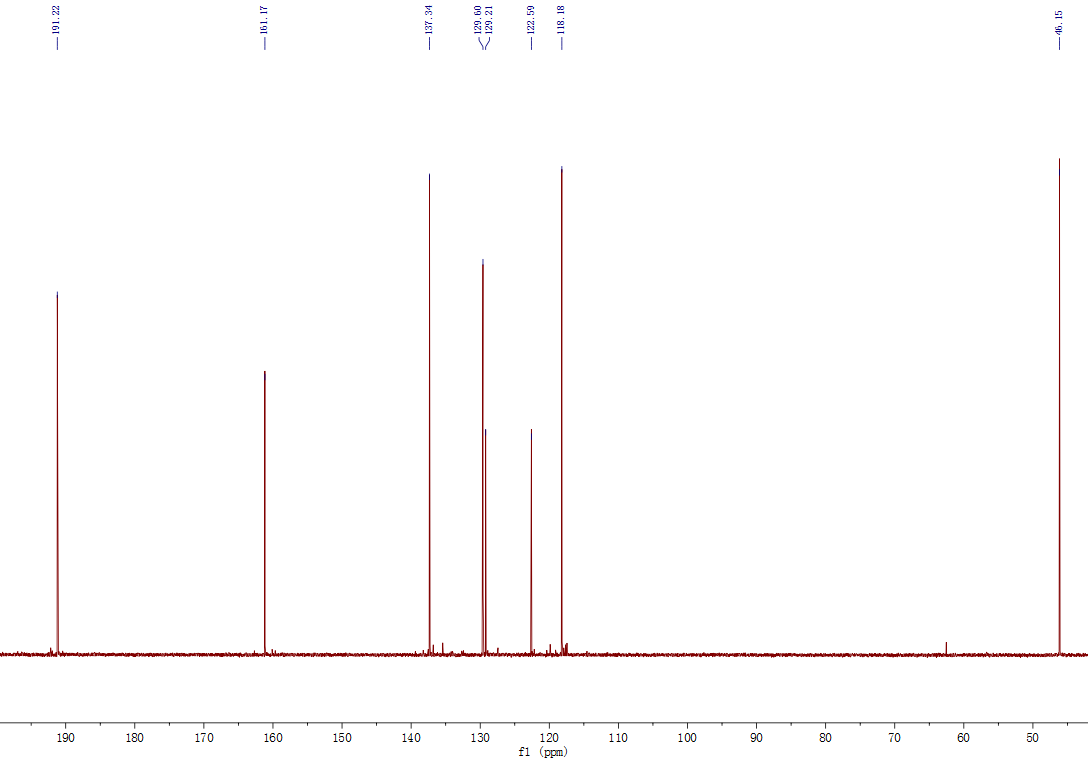


**Fig. S2** ^13^C NMR spectrum (151 MHz, d_6_-DMSO, 293 K) of **b**.

**c** A solution of acetic acid (22 ml, 50%) and hexamethylenetetramine (4.0 g) was reflexed at the progress of heating until the solid in these mixture completely dissolved and then recovered to room temperature. Added **b** (3.80g, 22mmol) and concentrated hydrochloric acid (50 mL) to the solution and refluxed it about 2 hours. Stop this reaction and put the reaction flask in refrigerator to gain pale yellow precipitate. These precipitate needed to be washed by water and finally dried and we gained **c** (4.41g).m.p = 106-108℃. ^1^H NMR (600 MHz, d_6_-DMSO) δ 11.76 (s, 1H), 10.32 (s, 1H), 9.87 (s, 1H), 8.19 (d, J = 2.2 Hz, 1H), 8.00 (dd, J = 8.6, 2.2 Hz, 1H), 7.15 (d, J = 8.6 Hz, 1H). ^13^C NMR (151 MHz, d_6_-DMSO) δ 191.49 , 190.48 , 165.87 , 136.11 , 132.43 , 128.81 , 122.99 .


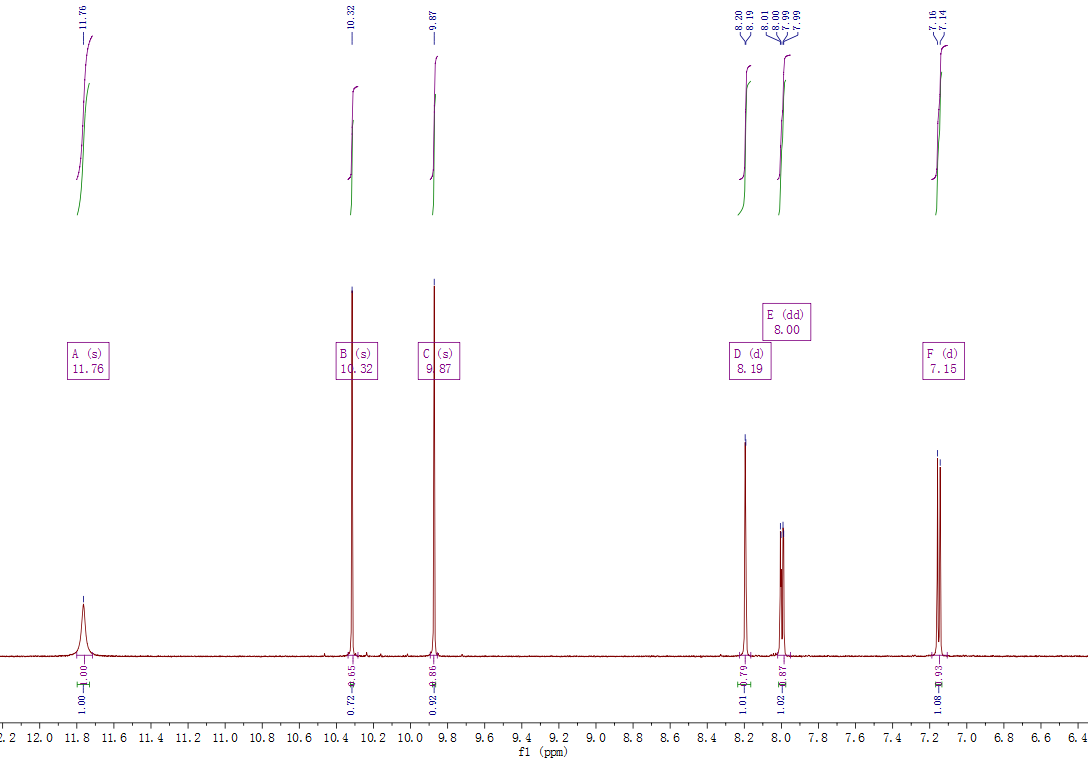


**Fig. S3** ^1^H NMR spectrum (600 MHz, d_6_-DMSO, 293 K) of **c**.


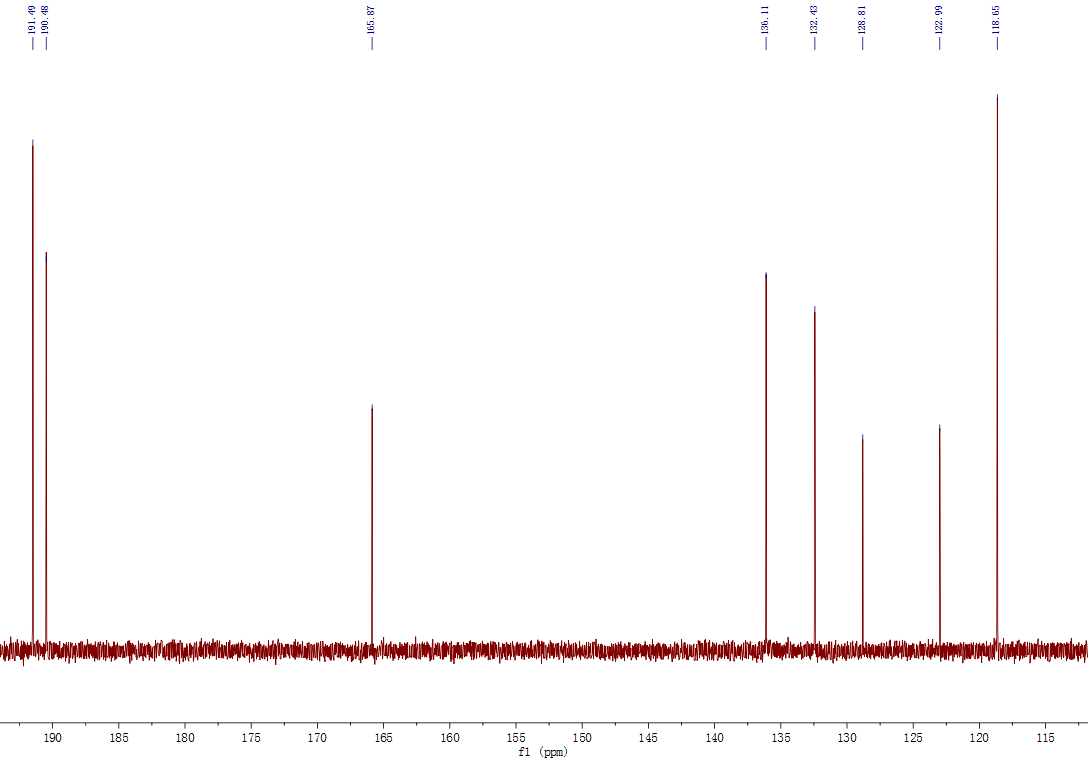


**Fig. S4** ^13^C NMR spectrum (151 MHz, d_6_-DMSO, 293 K) of **c**.

**L** Added 4-hydroxyisophthalaldehyde (2.94g, 20 mmol), 2-(hydrazonomethyl)phenol (5.71g, 42mmol) and several drops of acetic acid to ethanol as a reaction mixture. Stirred this mixture at 80℃ for 12 hours in a dry flask. After the reaction was complete, the reaction solution was evaporated under reduced pressure and extracted with ethyl acetate.The residue was purified by column chromatography on silica gel using progressively more polar 50:1 to 30:1 petroleum ether/ethyl acetate as the mobile phase to give compound **L** as a buff powder (3.09 g, m.p 270 ~ 273℃ ). ^1^H NMR (600 MHz, d_6_-DMSO) δ 11.52 (s, 1H), 11.34 (s, 1H), 11.10 (s, 2H), 9.03 (d, J = 4.8 Hz, 1H), 8.93 (s, 1H), 8.76 (s, 1H), 8.29 (s, 1H), 7.95 (d, J = 7.7 Hz, 1H), 7.90 (d, J = 8.6 Hz, 1H), 7.73 (d, J = 2.6 Hz, 2H),7.67 (d, J = 3.2 Hz, 1H), 7.66 (s, 1H), 7.39 (d, J = 7.2 Hz, 2H), 7.10 (d, J = 8.4 Hz, 1H), 6.97 (s, 4H).^13^C NMR (151 MHz, d_6_-DMSO) δ 163.44 , 162.97 , 161.94 , 161.36 , 159.09 , 133.58 , 133.27 , 131.68 , 131.27 , 120.04 , 119.56 , 118.69 , 116.87 . The [**L** - H^+^]^-^ peak appeared at 385.1021. which is coinciding well with that for the species [C_22_H_17_N_4_O_3_ - H]^-^ (m/z = 385.1345).


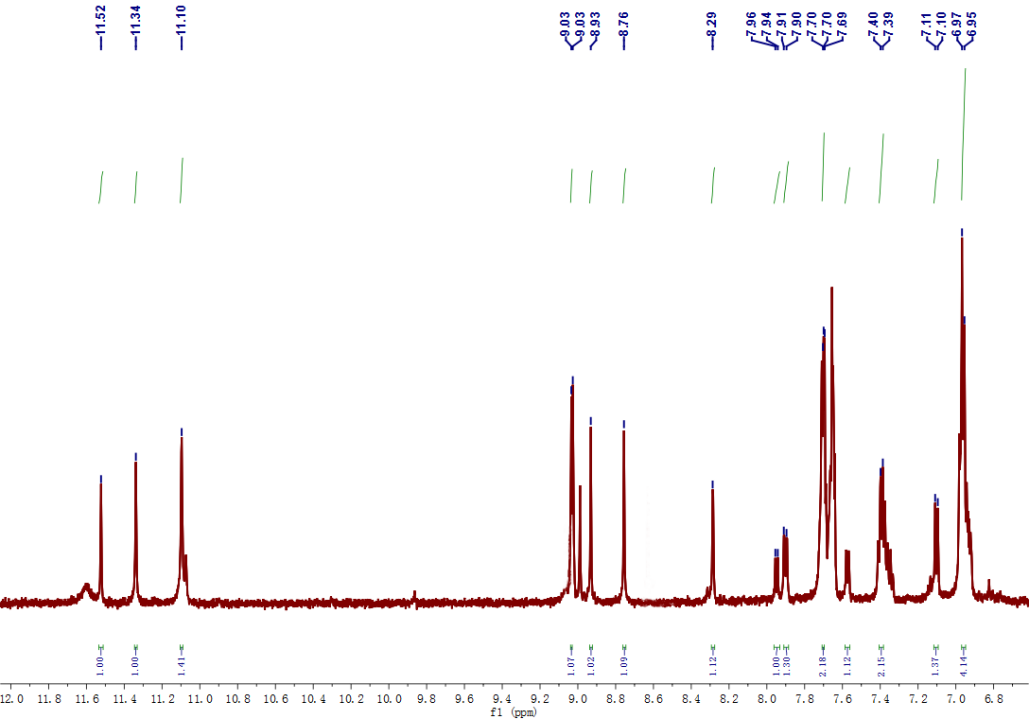


**Fig. S5** ^1^H NMR spectrum (600 MHz, d_6_-DMSO, 293 K) of **L**.


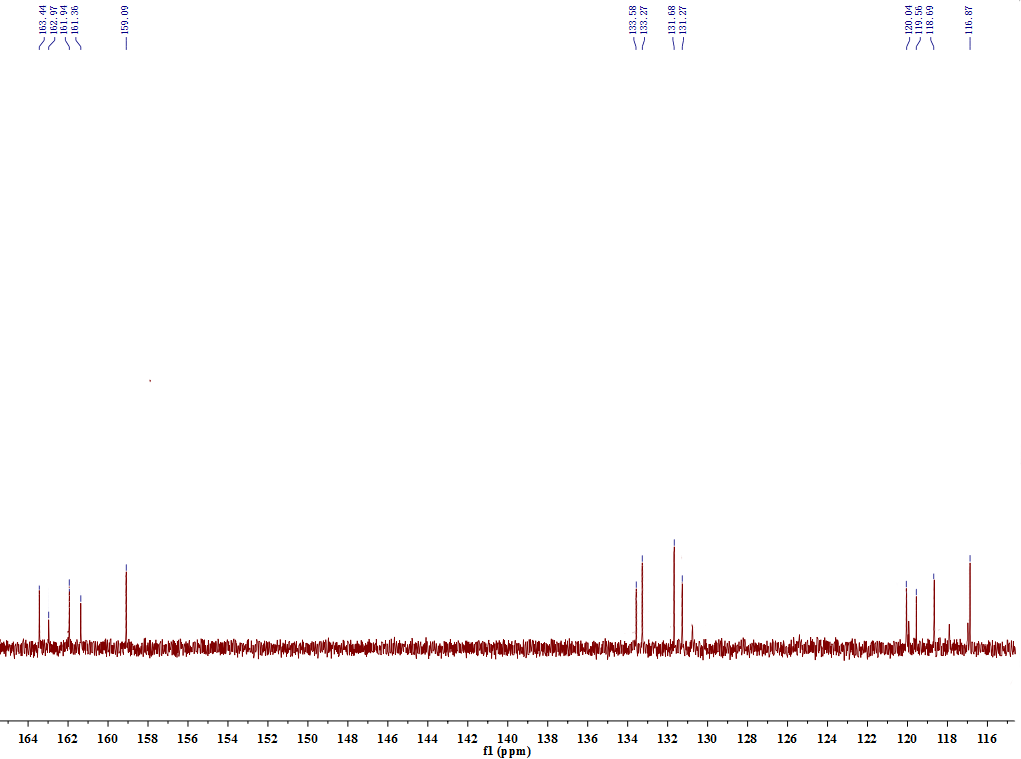


**Fig. S6** ^13^C NMR spectrum (151 MHz, d_6_-DMSO, 293 K) of **L**.


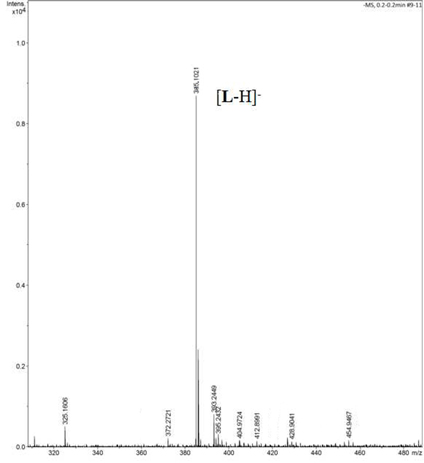


**Fig. S7**  ESI-MS spectrum of compound **L**.


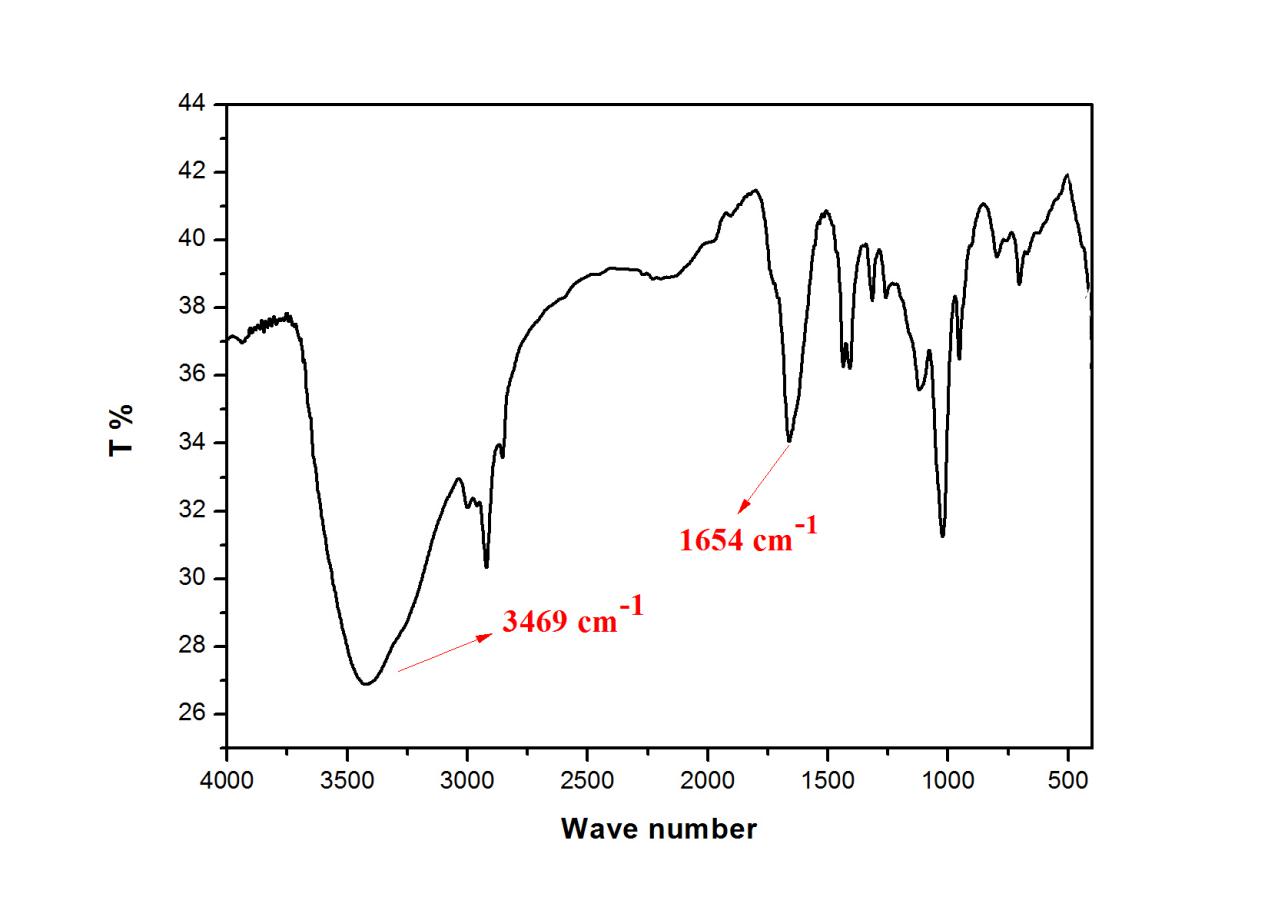


**Fig. S8**: IR spectra of **L** showing sharp peak at 1654 cm^-1^ for C=N bond and the broad peak at 3469 cm^-1^for -OH.


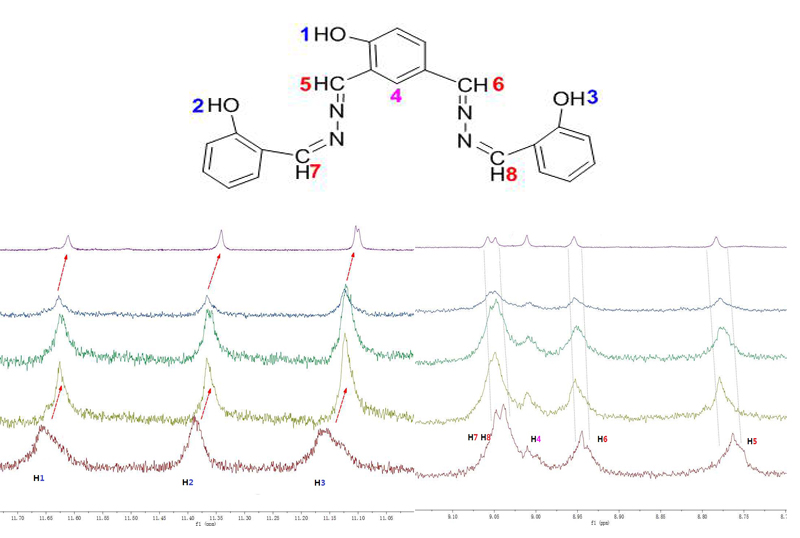


**Fig. S9** Partial ^1^H NMR titration spectra (d6-DMSO, 298 K, 400 MHz) of **L** (15mM) upon addition of Zn^2+^.


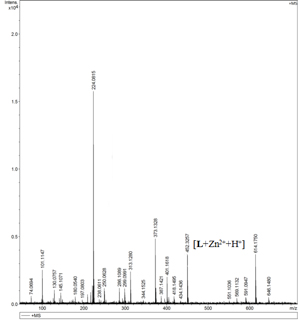


**Fig. S10** ESI-MS spectrum of compound **L** and Zn^2+^.

**Fig. S11** fluorescence titration spectra of **L** (c = 2 × 10^-5^ M) in the presence of different concentrations of Cd^2+^ ions in DMSO/H_2_O ~ HEPES buffer (80/20, v/v; pH = 7.23) solutions.

**Fig. S12** Fluorescence intensity at 524 nm of **L** versus the number of equiv. of Zn^2+^ added.
